# Histamine Produced by Gram-Negative Bacteria Impairs Neutrophil's Antimicrobial Response by Engaging the Histamine 2 Receptor

**DOI:** 10.1159/000525536

**Published:** 2022-07-20

**Authors:** Karim Dib, Amal El Banna, Clara Radulescu, Guillermo Lopez Campos, Gerard Sheehan, Kevin Kavanagh

**Affiliations:** ^a^Wellcome-Wolfson Institute for Experimental Medicine, Queen's University Belfast, Belfast, UK; ^b^Department of Biology, Maynooth University, Maynooth, Ireland

**Keywords:** Neutrophils, Phagocytosis, Histamine, Histamine receptors

## Abstract

We found that histamine (10^−9^ M) did not have any effect on the *in vitro* capture of *Escherichia coli* by neutrophils but accelerated its intracellular killing. In contrast, histamine (10^−6^ M) delayed the capture of *Escherichia coli* by neutrophils and reduced the amounts of pHrodo zymosan particles inside acidic mature phagosomes. Histamine acted through the H_4_R and the H_2_R, which are coupled to the Src family tyrosine kinases or the cAMP/protein kinase A pathway, respectively. The protein kinase A inhibitor H-89 abrogated the delay in bacterial capture induced by histamine (10^−6^ M) and the Src family tyrosine kinase inhibitor PP2 blocked histamine (10^−9^ M) induced acceleration of bacterial intracellular killing and tyrosine phosphorylation of proteins. To investigate the role of histamine in pathogenicity, we designed an *Acinetobacter baumannii* strain deficient in histamine production (hdc::TOPO). *Galleria mellonella* larvae inoculated with the wild-type *Acinetobacter baumannii* ATCC 17978 strain (1.1 × 10^5^ CFU) died rapidly (100% death within 40 h) but not when inoculated with the *Acinetobacter baumannii* hdc::TOPO mutant (10% mortality). The concentration of histamine rose in the larval haemolymph upon inoculation of the wild type but not the *Acinetobacter baumannii* hdc::TOPO mutant, such concentration of histamine blocks the ability of hemocytes from *Galleria mellonella* to capture *Candida albicans in vitro*. Thus, bacteria-producing histamine, by maintaining high levels of histamine, may impair neutrophil phagocytosis by hijacking the H_2_R.

## Introduction

Neutrophils are the most abundant white blood cells and constitute the first line of defense against bacterial and fungal pathogens [1]. These cells have developed a broad range of weapons to kill microorganisms, which include production of reactive oxygen species (ROS) and release of microbicidal granule constituents (degranulation) at the site of inflammation, in response to chemoattractants, proinflammatory cytokines, or bacterial motifs. Production of ROS and degranulation are also integral parts of phagocytosis, a complex process by which neutrophils (and other phagocytic cells) capture, engulf, and kill pathogens inside intracellular phagosomes. The capture of pathogens is facilitated by opsonization, a process by which microorganisms are coated with complement proteins or antibodies. In neutrophils, the main receptor binding complement-opsonized microorganisms is the β2 integrin Mac-1 [2].

Histamine is a major regulator of the immune response because most, if not all, immune cells respond to histamine through engagement of histamine receptors (H_1_R-H_4_R). Histamine is produced by the decarboxylation of histidine by histidine decarboxylases (HDCs) [3].

There is evidence in the literature which supports the view that neutrophils produce histamine at the site of inflammation. This is best exemplified in a study [3] showing that the mature 74 kDa form of HDC is not present in peripheral blood murine neutrophils, and the post-translational processing of the 53 kDa precursor form is only observed in neutrophils infiltrated into the mouse peritoneal cavity. Another study found that tracheobronchitis and pneumonia, caused by mycoplasma infection in mice, lead to biosynthesis of histamine by airways neutrophils, thereby contributing to lung inflammation [4]. Production of histamine by neutrophils is triggered by interaction with pathogens. Thus, neutrophils produce histamine *in vitro* when exposed to the Gram-negative bacterium *Pseudomonas aeruginosa* PAO1 strain [5] or the TLR_4_ ligand LPS [6, 7]. Similarly to human neutrophils, hemocytes (a population of innate immune cells in invertebrates that include phagocytic cells) from the tunicate *Styela plicata* produce histamine when exposed to different pathogen-associated molecular patterns [8]. This implies that histamine biosynthesis by phagocytic cells has been preserved through evolution from invertebrates to mammals thus demonstrating the fundamental role that the diamine plays in the regulation of the innate immune response of the host.

Production of histamine by neutrophils is intriguing and may imply autoregulation of neutrophil functions. This scenario is plausible as neutrophils express two histamine receptors, the H_2_R and the H_4_R [9]. The H_4_R and the H_2_R have a high and low affinity for histamine, respectively. Thus, the H_4_R and the H_2_R receptors are activated by nanomolar or micromolar concentrations of histamine, respectively [10]. The fact that neutrophils express two pharmacological distinct histamine receptors, with different affinities for histamine, may indicate that histamine has a dual role. Thus, depending on its concentration, histamine could either activate inflammatory functions of neutrophils (by engaging the H_4_R) to facilitate the killing of microorganisms or impair inflammatory functions (by engaging the H_2_R) to reverse and resolve the inflammatory process.

Neutrophils are not the only source of histamine during periods of infection. Indeed, it was shown that acute exacerbations of chronic bronchitis, cystic fibrosis, and pneumonia are associated with Gram-negative bacteria synthesizing histamine. Thus, *Pseudomonas aeruginosa*, a Gram-negative bacterium that is also a significant cystic fibrosis pathogen, as well as *Branhamella catarrhalis* and *Haemophilus parainfluenzae* synthesize clinically important concentrations of histamine [11].

It is not known why some Gram-negative bacteria produce histamine. Bacteria-derived histamine could represent a previously unappreciated evolutionarily conserved molecular dialog between host and pathogen, whereby production of histamine would regulate neutrophil phagocytosis and inflammatory functions to the advantage of the bacteria. In this study, we aimed to evaluate the role of both the H_4_R and H_2_R receptors in the capture and killing of bacteria by neutrophils. We also investigated the contribution of histamine produced by the Gram-negative bacteria *Acinetobacter baumannii* (*A. baumannii*) for pathogenicity in the *Galleria mellonella* (*G. mellonella*) larvae model of infection. We propose a model in which the H_4_R and H_2_R have opposite effects in terms of regulation of bacterial capture and killing by neutrophils and how histamine produced by bacteria could hijack the neutrophil response.

## Materials

Histamine and famotidine (ref: F6889) were purchased from Sigma-Aldrich/Merck. Ficoll-Hypaque was from GE Healthcare/Cytiva and Dextran 500 from Pharmacosmos (Denmark). The H_4_R antagonist JNJ 7777120 (ref: ab144405) was purchased from Abcam (UK). The PCR purification kit and the MiniPrep kit for plasmid isolation were from QiAgen (Germany). Zero Blunt^TM^ TOPO^TM^ PCR cloning Kit, with pCR^TM^-Blunt II-TOPO^TM^ Vector, One shot^TM^ TOP10 chemically competent *E. coli*, and pHrodo Red Zymosan A particles were purchased from ThermoFischer (UK).

The following antibodies were from cell signaling: the secondary HRP-conjugated anti-mouse or anti-rabbit antibodies (7074S and 7076S) and the anti-MMP-9 Ab (Ref: 3852S). The rabbit anti-VASP Ab (Ref: ab 229624) was from Abcam (Cambridge, UK); the anti-phosphotyrosine (Ref: 05-321X, clone 4G10) was from Millipore (UK). Forskolin (Ref: F 6886), IBMX (Ref: 15879), gentamicin, PP2, and SU6656 were purchased from Merck. The histamine ELISA kit was from IBL International GMBH (Hamburg, Germany). Histamine (Ref: H7125), N-formyl-methionyl-leucyl-phenylalanine (fMLP) (Ref: F3506), human lactoferrin (Ref: L0520), and anti-lactoferrin Abs (ref: L3262) were from Sigma/Aldrich/Merck. The protease inhibitor tablets were from Roche (Germany). The MACSxpress whole blood neutrophil isolation kit (Ref: 130-104-434) was from Miltenyi Biotec (Bergisch Gladbach, Germany). Protein assays were performed using the Bio-Rad (CA, USA) solutions and protocol (Ref: 500-0114).

## Methods

### Isolation of Human Neutrophils

Venous blood was collected from healthy donors by venous puncture in vacutainer EDTA blood collecting tubes. For the phagocytosis studies, neutrophils were isolated from the blood using the Dextran sedimentation and centrifugation through Ficoll-Hypaque [12]. For killing and capture assays, the cells (97% purity) were resuspended in RPMI medium supplemented with 20 mM HEPES; pH 7.4 (modified RPMI) at a concentration of 10^7^ cells/mL.

For the signaling studies, neutrophils were purified using the MACSxpress whole blood neutrophil isolation kit by following the instructions of the manufacturer. The cells were resuspended in HBSS medium supplemented with 1 mM MgCl_2_, 1 mM CaCl_2_, and 20 mM Hepes, pH 7.4 (modified HBSS). Cells were either pretreated with the H_4_R antagonist JNJ 7777120 or the H_2_R antagonist famotidine prior to stimulation with histamine.

### Cell Culture

Undifferentiated PLB-985 cells were grown at 37°C in an atmosphere of 5% CO_2_ in RPMI medium supplemented with 10% FCS to a density of 5 × 10^5^ cells/mL. Differentiation into neutrophil-like cells was carried out by culturing undifferentiated PLB-985 cells for 5 days in the RPMI medium supplemented with 5% FCS and 1.25% DMSO [13]. Cells were then collected by centrifugation (190 *g*, 10 min), washed in HBSS medium, and finally resuspended at a density of ∼1 × 10^6^ cells/mL in modified HBSS medium.

### Neutrophil Capture and Killing Assays

These assays were adapted from[14]. *E. coli* was inoculated in Luria Bertani (LB) broth and incubated overnight at 37°C in a flask placed in an orbital shaker. Bacteria were then diluted 1/10 and grown further for 1.5 h to reach the exponential phase. Bacterial concentrations were adjusted to 10^7^ CFU/mL. Bacteria in LB medium were spun down (2,500 *g*, 10 min), washed once with modified RPMI medium (RPMI medium supplemented with 20 mM Hepes pH 7.4), and then resuspended in modified RPMI medium containing 10% human serum (a mixture of five different serum samples). After 15 min, bacteria were spun down, washed with modified RPMI medium, and resuspended in the same buffer at a density of 10^7^ CFU/mL.

An equal volume of neutrophils (10^7^ cells/mL) and bacteria (10^7^ cells/mL) (1 mL of each) were mixed together in 15 mL conical tubes. After 10 or 30 min incubation at 37°C under shaking condition, 6 mL of cold-modified RPMI medium was added and the tubes were placed on ice. The tubes were spun (190 *g*, 10 min) in a cold centrifuge to pellet the neutrophils. The supernatant was collected and the number of *E. coli* remaining in the supernatant was estimated by serially diluting and colony counting on LB-agar plates. To this end, 20 μL of the serial diluted supernatants (in phosphate buffered saline, PBS) were spread onto LB agar plates (each plate is divided in four equal sections) and the plates were put in an incubator for 18 h at 30°C. The number of colonies formed on the plates was then counted and concentrations of bacteria were calculated by taking into account the dilution factors. The number of bacteria at time 0 (before addition of neutrophils) and time 30 min were also determined. Final concentrations of bacteria (CFU/mL) are corrected for bacterial growth. To test the effect of histamine on *E. coli* capture, the neutrophils (10^7^ cells/mL) were preincubated for 5 min at 37°C with histamine (10^−9^ M or 10^−6^ M) before addition of the bacterial suspension. When the protein kinase A (PKA) inhibitor H-89 (10 μM) was used, it was added 20 min prior to the addition of histamine (see above).

For intracellular killing assays, equal volumes of neutrophils (10^7^ cells/mL) and bacteria (10^7^ cells/mL) (1 mL of each) were mixed together in 15 mL conical tubes, which were placed on a shaker at 37°C. After 10 min or 30 min incubation, 6 mL of cold-modified RPMI medium was added. The tubes were spun (190 *g*, 10 min) in a cold centrifuge to pellet the neutrophils. The supernatants were discarded and the pellet washed three times with ice-cold-modified RPMI. The pellets were then resuspended in 1 mL of ice-cold-modified RPMI and saponin (0.1%) was added. After vortexing to disrupt neutrophils, the supernatants were diluted in PBS and the number of colonies were then measured as described above. The Src family tyrosine kinase inhibitors PP2 (5 μM) or SU6656 (5 μM) were added 20 min prior to the addition of histamine (see above).

For the gentamicin protection assay [15], neutrophils (10^7^ cells/mL) and bacteria (10^7^ cells/mL) (1 mL of each) were mixed in 15 mL conical tubes which were placed on a shaker at 37°C. After 10 min, 10 mL of cold-modified RPMI medium was added and the tubes were put on ice. The tubes were subjected to centrifugation (190 *g*, 10 min). The pellets were recovered, washed once with 5 mL cold-modified RPMI, and resuspended in the same medium. Thereafter, gentamicin (5 μg/mL) was added and the samples split into two. One sample received histamine (10^−9^ M), the other sample only vehicle (H_2_O). After 10 min or 30 min incubation, the cell suspension was subjected to centrifugation (190 *g*, 10 min), the pellet washed once with 10 mL PBS, and then resuspended in 1 mL PBS. Saponin was added (0.1%) followed by vortexing, and finally the number of colonies were measured as described above.

For the measurement of pHrodo zymosan particles capture by neutrophils, the following protocol was used: 100 μL of a neutrophil solution (10^6^/mL) in modified HBSS was placed in 96-well plates coated with fibrinogen [12], together with 100 μL of serum-opsonized red pHrodo zymosan A particles (as recommended by the manufacturer). TNF-α (20 ng/mL) and histamine (10^−9^ M or 10^−6^ M) were added or not to the wells, after which the plates were subjected to centrifugation (1 min, 190 *g*) to synchronize phagocytosis. The plates were then placed in the microplate reader at 37°C and the fluorescence was read over time (excitation 560 nm, emission 585 nm). Autofluorescence was substracted from each assay values. All assays were carried out in triplicates.

### Western Blot Analysis

Neutrophils, or differentiated PLB-985 cells, resuspended in modified HBSS medium (1 × 10^6^ cells in 1 mL), were pretreated or not with PP2 (5 μM), H-89 (10 μM), JNJ 7777120 (10^−8^−10^−5^ M) or famotidine (10^−8^−10^−5^ M) for 20 min at 37°C, after which histamine (10^−9^−10^−5^ M) was added to the samples. After 1 min, the reaction was stopped by adding 0.5 mL cold-modified HBSS and the tubes were put on ice. Controls cells only received vehicle (0.01% DMSO). The tubes were spun-down in a cold centrifuge (190 *g*, 10 min), and the supernatants were discarded. The pellets were lysed by adding 400 μL of ice-cold lysis buffer (100 mM Tris-HCl, pH 7.5, 1% Triton X-100, 5 mM EDTA, 5 mM EGTA, 50 mM NaCl, 5 mM NaF, 1 mM Na_3_VO_4_, and a protease inhibitor tablet). Cell lysates were clarified by centrifugation (10 min, 15,000 *g*) at 4°C, and 100 μL of 5X Laemmli buffer containing 1 mM DTT was added to the clarified supernatants. The samples (40 μg) were subjected to 8% SDS-PAGE and transferred to nitrocellulose membranes. The membranes were extensively washed under a running tap of distilled water to remove any traces of acrylamide and were then stained with Ponceau S to verify equal loading in each well prior to immunoblotting. Next, the membranes were blocked for 1 h in TBS buffer, supplemented with 0.2% Tween 20 (TBS-T) and 5% skimmed milk, and then incubated overnight at 4°C with an anti-VASP Ab (1 μg/mL dilution). After three 5 min washes with TBS-T buffer, the membranes were subsequently incubated for 1 h with goat peroxidase-conjugated anti-rabbit IgGs (1:2,500). The blots were again washed with TBS-T and Ab binding was visualized by enhanced chemiluminescence (ECL) using the GBOX ChemiXRQ. The ECL solution was prepared by mixing 6 mL of 0.1 M Tris pH 8.5, with 60 μL of 125 mM luminol (5-amino-2,3-dihydro-1,4-ptalazinedione) and 30 μL of 68 mM p-coumaric acid, and 3 μL of 30% H_2_O_2_ solution. Stocks of luminol solution and p-coumaric acid (all in DMSO) were kept at −20°C.

For the detection of tyrosine phosphorylated proteins, the cells were resuspended in HBSS-M medium at a density of 0.2 × 10^6^/mL and then treated as described above. The cells (1 × 10^6^ in 5 mL) were incubated in 15 mL tubes and the reactions stopped by adding 10 mL of cold HBSS-M. The cell pellets recovered after spinning down the tubes at 190 *g* for 10 min, were lysed with 0.4 mL of ice-cold lysis buffer (100 mM Tris-HCl, pH 7.5, 1% Triton X-100, 0.1% SDS, 5 mM EDTA, 5 mM EGTA, 150 mM NaCl, 5 mM NaF, 2 mM Na_3_VO_4_, and a protease inhibitor tablet). Hundred microliter of 5 × Laemmli buffer containing 1 mM DTT was added to the clarified samples. The samples (40 μg) were subjected to 10% SDS-PAGE and transferred to nitrocellulose membranes. The membranes were stained with Ponceau S to verify equal loading in each well prior to immunoblotting. The membranes were blocked for 1 h in TBS-T buffer and 5% BSA (fraction V) and then incubated overnight at 4°C with an anti-phospho-tyrosine Ab (1 μg/mL dilution). After three 5 min washes with TBS-T buffer, the membranes were subsequently incubated for 1 h with goat peroxidase-conjugated anti-mouse IgGs (1:2,500). The blots were again washed and antibody binding was visualized by ECL as described above.

For the detection of MMP-9 and lactoferrin, neutrophils resuspended in modified RPMI at a density of ∼1 × 10^7^/mL, were pretreated or not with histamine (10^−6^ M) for 5 min at 37°C. Thereafter, opsonized *E. coli* cells (1 × 10^7^/mL) were added (0.5 mL of neutrophil suspension and 0.5 mL of bacterial suspension) for different time periods. The reaction was stopped by adding 0.5 mL of ice-cold-modified RPMI and putting the tubes on ice. The tubes were then spun (2,500 *g*, 10 min) in a refrigerated centrifuge, the supernatants (free of bacteria and neutrophils) collected and split into two. In one fraction (0.5 mL), 150 μL of 5 × Laemmli buffer containing 1 mM DTT was added, followed by 5 min boiling of the samples. Detection of MMP-9 was then carried out by Western blot analysis, as described above, using an anti-MMP-9 antibody. The other fraction is used for the determination of lactoferrin concentration using an ELISA [16].

### Design of the *A. baumannii* Hdc-Deficient Strain

The protocol by Aranda et al. [17] was followed to design a mutant deficient in histamine production. A 500-bp internal fragment of the *A. baumannii* ATCC 17978 hdc gene (homology region) was designed by PCR using the following primers: FW: GAGGATGATCGACAAAAGGTA, REV: TTGTGGTGATTGGAAAGACT. The PCR product was purified by using the QiA quick PCR purification kit (Qiagen). After purification and quantification, the 500 bp PCR product was cloned into the pCR-blunt-TOPO plasmid, by following the protocol provided by the manufacturer (ThermoFischer, UK). Insertion of the plasmid into one Shot Top10 chemically competent *E. coli* was carried out by using the heat shock method. After transformation, 100 μL of bacterial suspension was placed on LB-agar plates containing kanamycin (50 mg/mL). After 18 h, colonies were picked on plates and grown in LB medium containing kanamycin (50 mg/mL). Glycerol stocks were then prepared and frozen at −80°C. Plasmids preparations were also carried out using the QiAPrep Spin Miniprep Kit (Qiagen). Thereafter, purified plasmids were introduced into competent *A. baumannii* ATCC 17978 strain by electroporation using the gene Pulser X cell electroporation systems (Bio-rad). Recombinant *A. baumannii* hdc::TOPO mutants, with a disrupted hdc gene, were selected on plates containing kanamycin (50 mg/mL) and grown overnight in LB medium supplemented with kanamycin (50 mg/mL). Genomic DNA was extracted from these kanamycin-resistant colonies and PCR was carried out to verify that the correct gene insertion had been obtained. To this end, two sets of primers were used for the generation of the amplicons. Set 1: FW: AATACGACTCACTATAGGG (T7 primer), Rev: CCATCATAAGGC­ATACGAC. Set 2: FW: AATACGACTCACTATAGGG (T7 primer), Rev: CATCATAAGGCATACGACAA.

The amplicons obtained by PCR were separated on 1% agarose gel and visualized. Their sizes were estimated in reference to molecular weight standards. The amplicons were further sequenced using the primers provided by the manufacturer in the kit.

### Determination of Histamine Concentration

Cultures of overnight grown *A. baumannii* ATCC 17978 or hdc::TOPO were adjusted to a OD value of 0.2 at 600 nm. An aliquot (10 μL) of the bacterial suspension was added to a 50 mL conical tube containing 10 mL of LB medium (or LB medium and kanamycin for hdc::TOPO) without or with histidine (10^−3^ M). The 10 mL suspensions are then transferred to a 250 mL conical flask. The flasks are put in an orbital shaker at 37°C. After 18 h, an aliquot (1 mL) of each bacterial suspensions is collected into 1.5 mL Eppendorf tubes and spun at 2,500 *g* for 10 min. Supernatants, free of bacteria, were collected and used for quantification of histamine using an ELISA kit by following the protocol provided by the manufacturer.

### Measurement of Bacterial Growth

*A. baumannii* wild-type (WT) or hdc::TOPO mutant strains were inoculated in LB medium and grown overnight in a glass flask under rotation at 37°C. Bacterial suspensions were diluted 1/100 in the LB broth (LB broth and kanamycin for hdc::TOPO mutant). The suspension for each strain was then dispatched into two flasks. Histidine (10^−3^ M final), was added in one flask, and vehicle (H_2_O) to the control flask. The flasks were shaken at 37°C and, after each hour, an aliquot (1 mL) is collected, transferred to a plastic cuvette, and the optical density at 600 nm is read. LB broth served as a reference. For later time points, aliquots were diluted in LB medium prior to reading OD values.

### *G. mellonella* Larvae Killing Assay

We used *G. mellonella* larvae with a weight between 250 and 350 mg in all assays. Ten randomly chosen larvae were used for each group. *A. baumannii* WT or hdc::TOPO mutant strains, in their exponential phase, were collected, washed in PBS, and then diluted to the indicated cell density, as determined by the optical density at 600 nm. Ten microliter of the inoculum was injected into the hemocoel of each larva through the last left proleg [18]. Bacterial colony counts on LB agar plates were used to confirm all inocula. Larvae were considered dead when they displayed no movement in response to puncture with a micro-needle.

### Hemocytes Preparation and *C. albicans* Capture Assays

Insect hemocytes were harvested from ten larvae of *G. mellonella*. Larval hemolymph was bled into 9 mL of insect physiological saline (IPS) containing 10 mM EDTA and 30 mM sodium citrate as anticoagulants. Hemocytes were pelleted by centrifugation (500 *g*, 5 min) at room temperature. The cells were washed once with IPS and finally resuspended in PBS containing 5 mM glucose [18].

*C. albicans* cells were opsonized using cell-free haemolymph diluted 1/10 in IPS. Phagocytosis was measured by incubating 1 × 10^7^ hemocytes with 2 × 10^6^*C. albicans* cells in a rapidly stirred chamber of a Clark type oxygen electrode at 37°C. Every 10 min, an aliquot was taken and the cell suspension was subjected to centrifugation (500 *g*, 10 min) to pellet the hemocyte population. An aliquot of the supernatant was diluted in YEPD broth and placed onto YEPD agar plates. After 18 h, the number of colonies was enumerated and the concentration of *C. albicans* in the supernatant was calculated by taking into account the dilution factor.

### Statistical Analysis

**Fig. 1:**
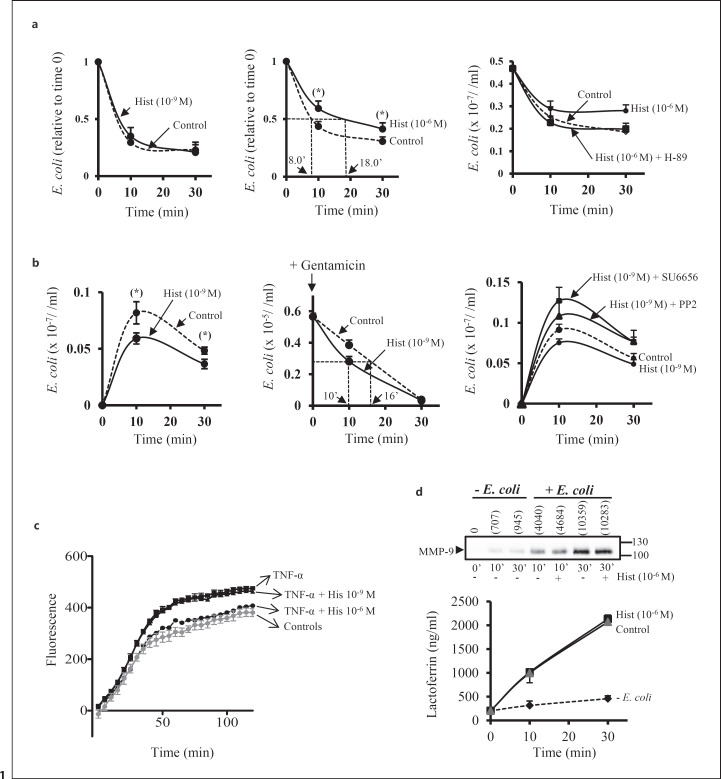
Histamine regulates the capture and killing of *E. coli* by neutrophils. **a** (left panel) Neutrophils (1 × 10^7^/mL) were preincubated without (dashed line) or with histamine 10^−9^ M (solid line) for 5 min. Thereafter, serum-opsonized *E. coli* cells (1 × 10^7^/mL) were added to the neutrophil preparation (1:1 ratio). After 10–30 min, the neutrophil/*E. coli* mixture was subjected to low speed centrifugation (190 *g*, 10 min) to pellet the neutrophils, but not the bacteria, and the supernatants were collected. The number of *E. coli* cells remaining in the supernatants was determined by colony counting on LB-agar plates. The data represent mean values ± SEM of three experiments using neutrophils from three different blood donors. **a** (middle panel) Neutrophils (1 × 10^7^/mL) were preincubated without (dashed line) or with histamine 10^−6^ M (solid line) for 5 min. Thereafter, the number of *E. coli* cells remaining in the supernatants, after pelleting neutrophils, was determined as described above. The data represent mean values ± SEM of seven experiments using neutrophils from seven different blood donors. **a** (right panel) Neutrophils (1 × 10^7^/mL) were pretreated with H-89 (10 μM, 20 min, solid square) or not pretreated with the PKA inhibitor (dashed line, and round circle) after which the cells were incubated or not with histamine (10^−6^ M) for 10–30 min. Thereafter, the number of *E. coli* remaining in the supernatant, free of neutrophils, was counted as described above. The data represent mean values ± SD of one experiment (out of two). **b** (left panel) Neutrophils (1 × 10^7^/mL) were preincubated without (dashed line) or with (solid line) histamine (10^−9^ M) for 5 min. Thereafter, serum-opsonized *E. coli* cells (1 × 10^7^/mL) were added (1:1 ratio). After 10–30 min, the neutrophil/*E. coli* mixture was subjected to low speed centrifugation (190 *g*, 10 min), and the recovered pellet was washed with PBS. The pellet was then resuspended in PBS and the number of *E. coli* cells was determined by colony counting. The data represent mean values ± SEM of five experiments using neutrophils isolated from five different blood donors. **b** (middle panel) Neutrophils (1 × 10^7^/mL) were incubated for 10 min with opsonized *E. coli* (1 × 10^7^/mL) after which the mixture was subjected to low speed centrifugation (190 *g*, 10 min). The pellets were washed with PBS, and then resuspended in modified RPMI containing gentamicin (5 mg/mL), in the absence (dashed lines), or presence (full line) of histamine (10^−9^ M). After 10 or 30 min incubation, the mixture was spun down, and the washed pellet resuspended in 1 mL PBS. The amount of *E. coli* cells associated with the pellet was then measured by colony counting. The data are mean values ± SEM of one representative experiment (out of two). Seven aliquots per assay were counted on LB-agar plates. **b** (right panel) Neutrophils (1 × 10^7^/mL) were pretreated with either PP2 (5 μM, 20 min, triangle) or SU6656 (5 μM, 20 min, square) after which the cells were incubated or not with histamine (10^−9^ M) for 5 min. Thereafter, serum-opsonized *E. coli* cells (1 × 10^7^/mL) were added (1:1 ratio). After 10–30 min, the neutrophil/*E. coli* mixture was subjected to low speed centrifugation, and the amount of *E. coli* associated with the pellet was counted as described in **b**, left panel. One experiment is shown (out of two). **c** Neutrophils (10^5^) were incubated on 96-well plates coated with fibrinogen in the absence (gray circles) or presence of TNF-α (20 ng/mL) (black square), or in the presence of TNF-α (20 ng/mL) and histamine (10^−9^ M) (black triangles) or TNF-α (20 ng/mL) and histamine (10^−6^ M) (black circles). Serum-opsonized pHrodo zymosan A particles were added to each wells. The fluorescence (585 nm) was read over time. The data represent mean values ± SD of triplicates in one representative experiment (out of two). **d** Neutrophils (1 × 10^7^/mL) were preincubated without or with histamine (10^−6^ M) for 5 min. Thereafter, serum-opsonized *E. coli* cells (1 × 10^7^/mL) were added to the neutrophil preparation (1:1 ratio). After 10–30 min, the neutrophil/*E. coli* mixture was subjected to low speed centrifugation (190 *g*, 10 min) to pellet the neutrophils, and the supernatants were collected. The level of MMP-9 in the supernatants was measured by Western blot analysis. The top panel depicts a representative Western blot; the position of pro-MMP-9 is indicated by an arrow on the left hand side. The graph represents the accumulation of lactoferrin in the supernatants from neutrophils pretreated (triangle) or not (square) with histamine (10^−6^ M), and exposed to *E. coli* cells. As a control, the release of lactoferrin from neutrophils not exposed to *E. coli* is shown (dotted line). The data represent mean values ± SEM of one representative experiment (out of two) performed in triplicates (for the lactoferrin data).

In Figure 1, a paired Student's *t* test was used to assess statistical difference between control cells and cells stimulated with histamine 10^−6^ M or histamine 10^−9^ M (*p < 0.05). In Figure 7, a Mantel-Cox test was used to compare the survival of *G. mellonella* treated with *A. baumannii* ATCC 17978 versus hdc::TOPO mutant. The statistical significance is given in Figure 7b.

### Densitometry Analysis

The densitometric analysis of the bands from Western blots was determined using the software Image J.JS.

## Results

To investigate a possible contribution of the H_4_R and/or the H_2_R in the capture and/or killing of bacteria by neutrophils, freshly isolated human neutrophils were incubated with serum-opsonized *E. coli*, either in the absence or presence of histamine. Two concentrations of histamine were used: 10^−9^ M (to engage the H_4_R) or histamine 10^−6^ M (to engage both the H_2_R and the H_4_R) [10]. After 10–30 min incubation, the mixture was subjected to centrifugation (190 *g*, 5 min) to pellet the neutrophils but not the bacteria. Then, the amount of *E. coli* remaining in the extracellular medium was determined by colony counting on LB-agar plates (extracellular bacteria). We found that the kinetics of *E. coli* capture by neutrophils were identical between nonstimulated control cells and neutrophils stimulated with histamine (10^−9^ M) (Fig. 1a, left panel). In contrast, in the presence of a high dose of histamine (10^−6^ M), we observed a significant delay in the capture of *E. coli* by neutrophils (Fig. 1a, middle panel). This is illustrated by the fact that the time required by neutrophils to capture half of the amount of bacteria added at time zero (normalized to one unit) was 8 min in the absence of histamine and 18 min in the presence of histamine (10^−6^ M). Thus, the low affinity H_2_R receptor, but not the H_4_R, negatively regulates pathogen capture by neutrophils. We also found that a pretreatment of neutrophils with the PKA inhibitor H-89 (10 μM) totally prevented histamine (10^−6^ M) to block *E. coli* capture (Fig. 1a, right panel).

We next investigated the possible role of the H_4_R in intracellular killing of bacteria. To test this, the neutrophil/*E. coli* mixture was incubated in the absence or presence of histamine (10^−9^ M) for 10–30 min. The mixture was then spun down (190 *g*, 10 min); the neutrophils were disrupted by the addition of saponin, after which the amount of *E. coli* associated with pelleted neutrophils was measured. A 10 min incubation time is optimum for the loading of neutrophils with *E. coli* [14]. After 10 min, the amount of *E. coli* associated with neutrophils declined thus illustrating that the captured pathogens are internalized and killed in phagosomes fused with granules containing antimicrobials. Interestingly, we found that the amount of *E. coli* associated with neutrophils was significantly (*p* < 0.05) lower when neutrophils were exposed to histamine (10^−9^ M) (Fig. 1b, left panel). Since histamine (10^−9^ M) had no effect on the capture of *E. coli* by neutrophils, and taking the fact that the H_2_R is not activated by nanomolar concentrations of histamine, our results indicate that engagement of the H_4_R leads to augmented intracellular killing of bacteria.

We also carried out gentamicin protection assays [15] to further prove the role of the H_4_R in the regulation of intracellular killing of microorganisms by neutrophils. The rationale for performing this assay is that gentamicin kills bacteria bound to the membrane surface of neutrophils but not internalized bacteria. To this end, neutrophils were incubated with *E. coli* for 10 min, after which the neutrophils were spun down, washed, and resuspended in RPMI medium containing gentamicin (5 μg/mL). The cell suspension was then split into two tubes. In one tube, histamine (10^−9^ M) was added. In the other tube, no histamine was added (control cells). Thereafter, the amount of *E. coli* associated with pelleted neutrophils was measured over time. We found that addition of histamine (10^−9^ M) accelerated the speed of *E. coli* killing by neutrophils. This is evidenced by the fact that control neutrophils kill half of the captured microorganisms in 16 min. In contrast, to accomplish the same task, neutrophils exposed to histamine (10^−9^ M) require 10 min (Fig. 1b, middle panel). When neutrophils were preincubated with the Src family tyrosine kinase inhibitors PP2 (pyrazolo-pyrimidie structure) or SU6656 (indolinone analog), acceleration of *E. coli* killing induced by histamine (10^−9^ M) was abolished (Fig. 1b, right panel).

We also measured the capture of pHrodo zymosan particles by adherent neutrophils. The rationale for using this pHrodo-based system is that it measures phagocytic activity based on acidification of particles as they are ingested. This means that fluorescence is emitted only when the particles are engulfed in mature acidic phagosomes. In neutrophils, after 10 min exposure to particles, the cells exhibit a slow phase of acidification which does not reverse by 1 h [19]. We found that the intensity of fluorescence was augmented with time in control neutrophils (gray circles) and reached a plateau at around 1 h. Stimulation of neutrophils with TNF-α augmented the number of particles inside acidic phagosome (black square) which is an indicative of activation of Mac-1-dependent capture of the particles. Pretreatment of neutrophils with histamine (10^−9^ M) did not modify the fluorescence intensity over time in cells stimulated with TNF-α. However, a pretreatment of neutrophils with histamine (10^−6^ M) reduced significantly the number of particles inside acidic phagosomes as evidenced by a decrease in fluorescence emission over time in TNF-α stimulated cells (Fig. 1c). In parallel, we investigated whether histamine (10^−6^ M) regulated neutrophil degranulation in response to *E. coli*. To this end, neutrophils and opsonized *E. coli* were incubated for 10–30 min (as described above) after which the amount of MMP-9 (a marker for specific and gelatinase granules) and lactoferrin (a marker for specific granules) in bacteria- and neutrophil-free supernatants were measured by Western blot analysis, or ELISA, respectively. By Western blot analysis, using an anti-MMP-9 Ab, we detected a protein in the supernatant of Mw ∼105 kDa which corresponds to the monomeric form of pro-MMP-9 [20]. As expected, we found low amounts of granule markers when neutrophils were not exposed to opsonized *E. coli* (basal conditions) (Fig. 1d). Addition of opsonized *E. coli* to neutrophils led to a time-dependent increase in the amount of both pro-MMP-9 and lactoferrin in the extracellular medium. However, a pretreatment of the cells with histamine (10^−6^ M) had no effect on *E. coli*-induced degranulation (Fig. 1d). This control experiment confirmed that histamine (10^−6^ M) delayed bacterial capture by neutrophils as histamine did not inhibit the release of microbicidal substances contained in neutrophil granules (which could have explained the augmented level of viable *E. coli*).

Together, our results demonstrate that in human neutrophils, the H_2_R and the H_4_R have two different functions in terms of capture and killing of microorganisms. The H_2_R negatively regulates the capture of *E. coli* by neutrophils, whereas activation of the H_4_R augments intracellular killing of pathogens.

### The H_2_R, but Not the H_4_R, Is Coupled to the cAMP/PKA Pathway

**Fig. 2:**
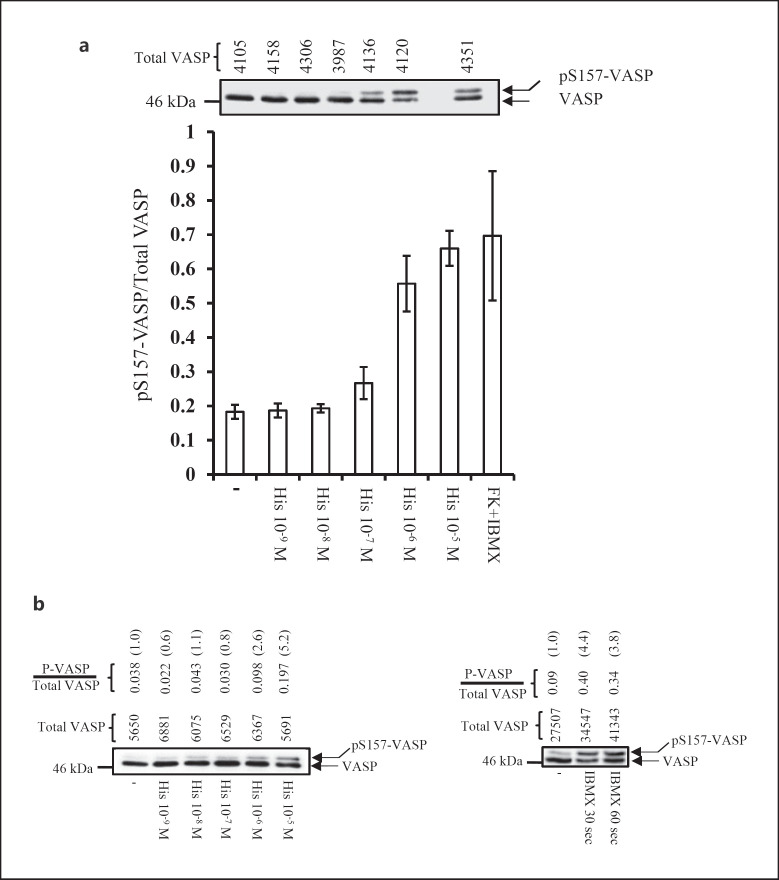
Dose-dependent effect of histamine on VASP phosphorylation on Ser 157 in neutrophils and differentiated PLB-985 cells. **a** PLB-985 cells differentiated into neutrophil-like cells, were stimulated with different concentrations of histamine (10^−9^−10^−5^ M), or a combination of FK (10^−5^ M) plus IBMX (10^−5^ M). After 1 min, the cells were lysed and solubilized in Laemelli buffer. Equal amounts of proteins (30 μg) were loaded in each well; proteins were separated by 8% SDS-PAGE, transferred to a nitrocellulose membrane and immunoblotted with an anti-VASP pAb. The top panel depicts a representative Western blot; the positions of pS157-VASP and VASP are indicated on the right hand side of the blot by arrows. The densitometry value of total VASP (used as a loading control) for each sample is indicated on the top of the representative blot. The diagram illustrates densitometric analysis of the pS157-VASP/total VASP ratio. The data represent means ± SD of 3 independent experiments. **b** (left panel) Neutrophils (1 × 10^6^) were stimulated with histamine (10^−9^−10^−5^ M) after which the pS157-VASP/total VASP ratio was determined as described in **a**. The panel depicts a representative Western blot (out of two experiments). The densitometry value of total VASP (used as a loading control) for each sample is indicated on the top of the representative blot. The pS157-VASP/total VASP ratio is also given. The amount of pS157-VASP/total VASP in control cells has been normalized to one. **b** (right panel) As a control, neutrophils were stimulated with IBMX for 30 s or 60 s after which the ration of pS157-VASP/total VASP was determined as described above. The densitometry value of total VASP (used as a loading control) for each sample is indicated on the top of the representative blot. The pS157-VASP/total VASP ratio is also given. A representative experiment out of two is shown. FK, forskolin.

We next investigated the signaling pathways activated by the H_2_R and the H_4_R in human neutrophils, as well as in PLB-985 cells differentiated into neutrophil-like cells. We used differentiated PLB-985 cells because these cells have remarkable similarities to human neutrophils in terms of signaling and functions [13, 21]. We investigated whether the H_2_R and/or the H_4_R were coupled to the production of cAMP and activation of PKA. To this end, differentiated PLB-985 cells (Fig. 2a), or human neutrophils (Fig. 2b), were incubated with histamine (10^−9^−10^−5^ M). After 1 min, the cells were lysed, cell lysates were separated on 8% SDS-PAGE, and proteins transferred to a nitrocellulose membrane. To assess cAMP production indirectly, we measured by Western blot the phosphorylation of the cytoskeletal protein Vasodilator-stimulated phosphoprotein (VASP) on Ser 157. In human neutrophils, phosphorylation of VASP occurs in response to fMLP, IL-8, or leukotriene B_4_. Such ligand-induced VASP phosphorylation is blocked by the PKA inhibitor H-89 and the adenyly cyclase inhibitor SQ22536. Thus, VASP phosphorylation on Ser 157 is catalyzed by the cAMP-dependent PKA [22]. Of interest, phosphorylation of VASP on Ser 157 leads to a change in electrophoretic mobility. Thus, it is easy to separate and detect both the Ser 157 phosphorylated and non-phosphorylated forms of VASP by Western blot analysis using an anti-VASP antibody [22]. We found that stimulation of differentiated PLB-985 cells (Fig. 2a), or human neutrophils (Fig. 2b, left panel), with high (10^−6^−10^−5^ M), but not low (10^−9^−10^−7^ M) doses of histamine, led to a significant increase in basal phosphorylation of VASP. With the highest concentration of histamine (10^−5^ M), we obtained 3.6- and 5.2-fold increases (over control cells) of the Ser 157 VASP/total VASP ratio in differentiated PLB-985 cells and neutrophils, respectively. Equal amount of proteins (∼30 μg) were loaded in each well of the gels. This was confirmed by quantifying the amount of total VASP (taken as loading controls) in each lane. To further ensure that VASP is phosphorylated in response to histamine, we normalized the densitometry values obtained for the Ser 157 VASP signal to the densitometry values of the corresponding total VASP (see above) [22].

As a control, we used IBMX and forskolin, which are commonly used to augment intracellular level of cAMP. We found that stimulation of differentiated PLB-985 cells with forskolin (10^−5^ M) and IBMX (10^−5^ M) (Fig. 2a), or human neutrophils with IBMX (10^−5^ M) (Fig. 2b, right panel), led to phosphorylation of VASP on Ser 157, similarly to what we found with histamine (10^−6^−10^− 5^ M).

**Fig. 3:**
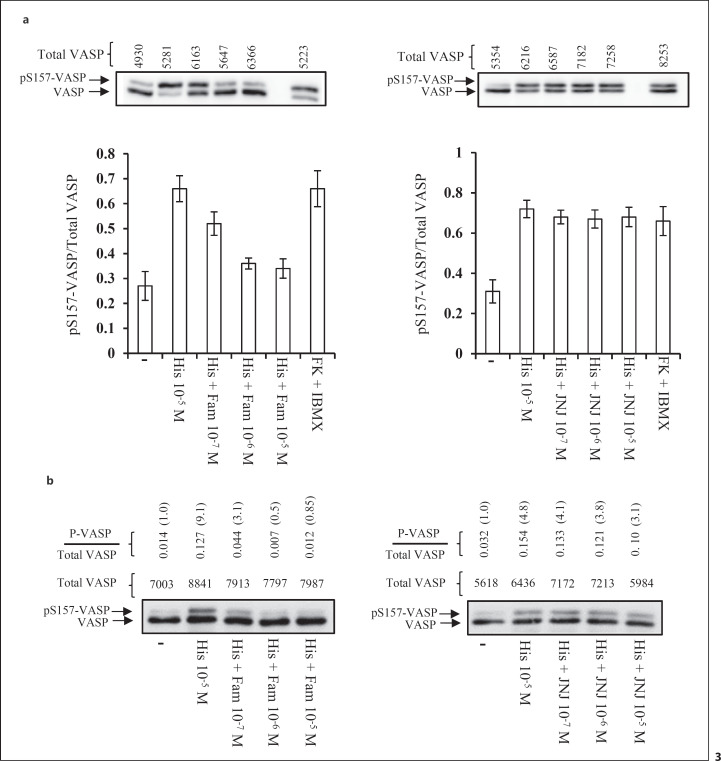
The H_2_R antagonist famotidine, but not the H_4_R antagonist JNJ777120, blocks histamine-induced phosphorylation of VASP on Ser 157. **a** PLB-985 cells differentiated into neutrophil-like cells, were pretreated for 20 min with either Fam (10^−7^−10^−5^ M, left panel) or JNJ 7777120 (JNJ, 10^−7^−10^−5^ M, right panel). Thereafter, the cells were stimulated with histamine (10^−5^ M) or a combination of FK (10^−5^ M) plus IBMX (10^−5^ M). After 1 min, the cells were lysed and the ratio of pS157-VASP/total VASP was determined as described in the legend to Figure 2. The top panels depict a representative Western blot. The diagram illustrates densitometric analysis of the pS157-VASP/total VASP ratio. The data represent means ± SD of 3 independent experiments. The densitometry value of total VASP (used as a loading control) for each sample is indicated on the top of the representative blot. **b** Neutrophils were pretreated for 20 min with either Fam (10^−7^−10^−5^ M, left panel) or JNJ 7777120 (10^−7^−10^−5^ M, right panel). Thereafter, the cells were stimulated with histamine (10^−5^ M) after which the pS157-VASP/total VASP ratio was determined as described above. The Western blot shown is representative of two experiments. The densitometry value of total VASP (used as a loading control) for each sample is indicated on the top of the representative blot. The pS157-VASP/total VASP ratio is also indicated on the top of the blot. The data have also been normalized to the amount of pS157-VASP/total VASP in control cells (taken as one). FK, forskolin; Fam, famotidine.

To confirm further that the H_2_R, but not the H_4_R, is coupled to the cAMP/PKA pathway, we made use of famotidine and JNJ 7777120, which are antagonists of the H_2_R and the H_4_R, respectively. We found that the phosphorylation of VASP on Ser 157 induced by histamine (10^−5^ M) was blocked in a dose-dependent manner by a pretreatment of PLB-985 cells (Fig. 3a) or neutrophils (Fig. 3b) with the H_2_R antagonist famotidine (10^−7^−10^−5^ M). In contrast, pretreatment of these cells with the H_4_R antagonist JNJ 7777120 (10^−7^−10^−5^ M) did not. All together, these results demonstrate that the H_2_R, but not the H_4_R, is coupled to the cAMP/PKA pathway.

### The H_4_R, but Not the H_2_R, Is Coupled to the Src Tyrosine Kinase Pathway

**Fig. 4:**
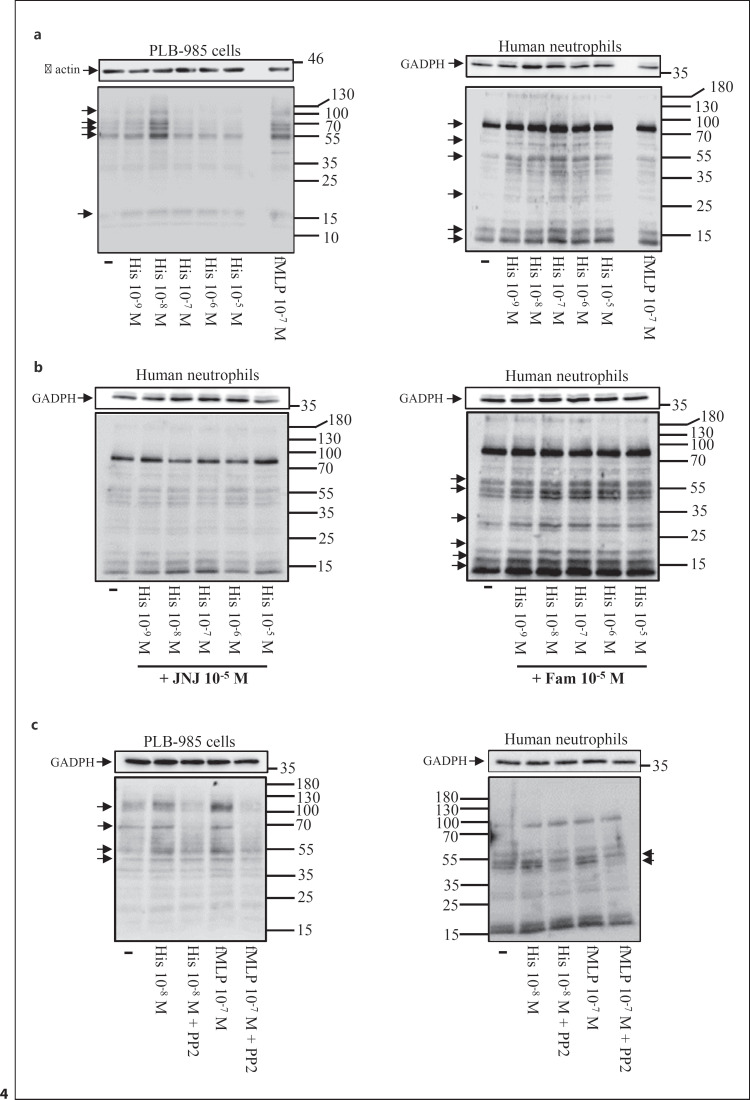
Histamine induces tyrosine phosphorylation of proteins in neutrophils. **a** PLB-985 cells differentiated into neutrophil-like cells (left panel) or neutrophils (right panel) were stimulated with histamine (10^−9^−10^−5^ M) or fMLP (10^−7^ M). After 1 min, the cells were lysed. Equal amount of protein lysates (40 μg) were loaded in each lane of the gels. Proteins were separated on 10% SDS-PAGE and transferred to a nitrocellulose membrane. The membrane was immunoblotted with an anti-PY mAb. The panel depicts a representative Western blot (out of three); the position of proteins phosphorylated in response to histamine is indicated on the right part of the blots by arrows. **b** Neutrophils were pretreated for 20 min with JNJ 7777120 (10^−5^ M) (left panel), or Fam (10^−5^ M) (right panel) after which the cells were stimulated with histamine (10^−9^−10^−5^ M). After 1 min, the cells were lysed and the tyrosine phosphorylation pattern was determined as described above. The panel depicts a representative Western blot (out of three); the position of proteins phosphorylated in response to histamine is indicated on the right part of the blot by arrows. **c** Differentiated PLB-985 cells (left panel) or neutrophils (right panel) were pretreated for 20 min with PP2 (5 μM) after which the cells were stimulated with histamine (10^−8^ M) or fMLP (10^−7^ M). After 1 min, the cells were lysed and the tyrosine phosphorylation pattern was determined as described above. At least 3 independent experiments were performed with human neutrophils and PLB-985 cells.

N-formulated peptides, such as fMLP, are potent agonists of neutrophil responses implicated in host defense [23]. Stimulation of neutrophils with fMLP triggers a rapid activation of tyrosine kinases of the Src family as well as Syk [23, 24, 25], such kinases are indispensable for the activation of inflammatory functions required for the intracellular killing of microorganisms. We next compared the pattern of tyrosine phosphorylated proteins in response to fMLP and histamine. We detected tyrosine phosphorylation of proteins in differentiated PLB-985 cells in response to low doses (10^−9^−10^−8^ M), but not high doses (10^−7^−10^−5^ M) of histamine (Fig. 4a, left panel). Furthermore, the pattern of tyrosine phosphorylated proteins, obtained in response to histamine (10^−9^−10^−8^ M), was similar to the one obtained in response to fMLP (10^−7^ M). The lack of tyrosine phosphorylation in response to high doses of histamine may be explained in two ways, either homologous desensitization of the H_4_R by histamine, or heterologous desensitization of the H_4_R triggered by engagement of the H_2_R as reported for other G protein-coupled receptors [26].

We also observed tyrosine phosphorylation of proteins in neutrophils stimulated with histamine (10^−9^−10^−5^ M) (Fig. 4a, right panel). Interestingly, a concentration of histamine as low as 10^−9^ M augmented basal tyrosine phosphorylation of proteins. The highest level of tyrosine phosphorylation was observed with histamine (10^−7^ M). Furthermore, a pretreatment of neutrophils with JNJ 7777120 (10^−5^ M) totally blocked tyrosine phosphorylation of proteins induced by histamine (10^−9^−10^−5^ M) (Fig. 4b, left panel). In contrast, a pretreatment of the cells with famotidine (10^−5^ M) did not (Fig. 4b, right panel). These results demonstrate that in human neutrophils and differentiated PLB-985 cells, the H_4_R, similarly to fMLP receptors, is coupled to Src/Syk family tyrosine kinases, a pathway controlling activation of neutrophil inflammatory functions. We further confirmed this assumption by showing that a pretreatment of differentiated PLB-985 cells (Fig. 4c, left panel) or neutrophils (Fig. 4c, right panel) with the Src family tyrosine kinase inhibitor PP2 totally blocked histamine- or fMLP-induced tyrosine phosphorylation of proteins.

### Design of a Hdc-Deficient *A. baumannii* ATCC 17978 Strain

**Fig. 5:**
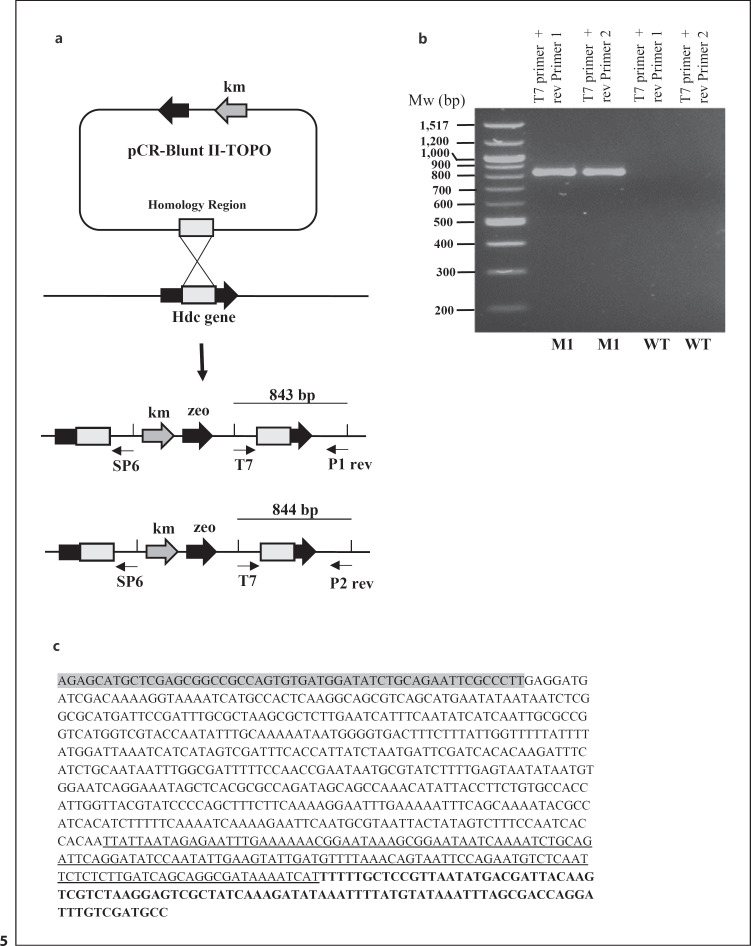
Construction of the hdc-deficient *A. baumannii* ATCC 17978 strain. **a** A region of the hdc gene (homology region) was amplified by PCR using the genomic DNA of *A. baumannii* ATCC 17978 as a template. The PCR product was purified, and inserted in to the pCR-Blunt II-TOPO plasmid. Competent *A. baumannii* ATCC 17978 were electroporated with the pCR-Blunt II-TOPO plasmid containing the homology region of the hdc gene. Recombinants were selected on kanamycin plates. The predicted organization of the genomic DNA of the hdc recombinant hdc::TOPO is shown. To confirm that the hdc gene has been disrupted, the region between the T7 promoter (plasmid sequence) and a region upstream of the hdc gene were amplified by PCR using genomic DNA of the hdc::TOPO recombinant as a template. Two reverse primers (P1 rev or P2 rev) recognizing a gene sequence upstream of the hdc gene were designed. The predicted sizes of the amplicons are 843 and 844 bp with P1 rev and T7 forward primers and P2 rev and T7 forward primers, respectively. **b** The amplicons obtained by PCR using either genomic DNA from the recombinant hdc::TOPO or the WT strain as templates were separated on 1% agarose gel and visualized. The size of the molecular standards (in bp) is indicated on the left hand side of the gel. **c** the sequence of the amplicon obtained by PCR using the P1 rev and T7 forward primers and genomic DNA of the hdc mutant hdc::TOPO as a template is indicated. The gray part is the plasmid sequence upstream of the T7 promoter, followed by the sequence of the homology region, then the sequence of the end part of the hdc gene (underlined), and finally the genomic sequence 3′ upstream of the hdc gene (bold).

To evaluate the contribution of histamine produced by Gram-negative bacteria in pathogenicity, we designed a hdc-deficient *A. baumannii* strain by gene disruption [17]. To confirm that the hdc gene was disrupted by plasmid insertion, regions between the T7 promoter (plasmid sequence) and upstream of the hdc gene were amplified by PCR using genomic DNA of *A. baumannii* hdc::TOPO mutant as a template. The expected calculated PCR fragment sizes are 843 bp (with P1 rev and T7 forward primers) and 844 bp (with P2 rev and T7 forward primers), respectively (Fig. 5a). The amplicons obtained by PCR were separated on 1% agarose gel and visualized. Their sizes were estimated to be ∼850 bp (Fig. 5b). Thus, the sizes of the PCR products matched the predicted sizes of the amplicons. As a control, we showed that no such amplicons were generated by PCR, using the same sets of primers, with genomic DNA of WT *A. baumannii* ATCC 17978 (Fig. 5b).

To confirm further hdc gene disruption, we sequenced one of the amplicon and its sequence matched the predicted sequence (Fig. 5c). The gray part of the amplicon sequence corresponds to the plasmid sequence upstream of the T7 promoter, followed by the amplicon sequence of the homology region cloned into the TOPO plasmid, then the amplicon sequence of the end part of the hdc gene (underlined), and finally the genomic sequence upstream of the hdc gene (bold).

### Functional Activity of the *A. baumannii* hdc::TOPO Mutant

To verify that hdc gene disruption had functional consequences, we compared histamine production by both the hdc::TOPO mutant and the WT strain. To this end, we incubated the strains in a glass flask containing LB medium, in the absence or presence of histidine (10^−3^ M). After 18 h under shaking conditions, supernatants, free of bacteria, were collected and histamine concentrations were measured by ELISA. The rationale for adding histidine to the LB medium is that this amino acid is a substrate for hdc and hdc of Gram-negative bacteria are inducible by histidine [27].

**Fig. 6:**
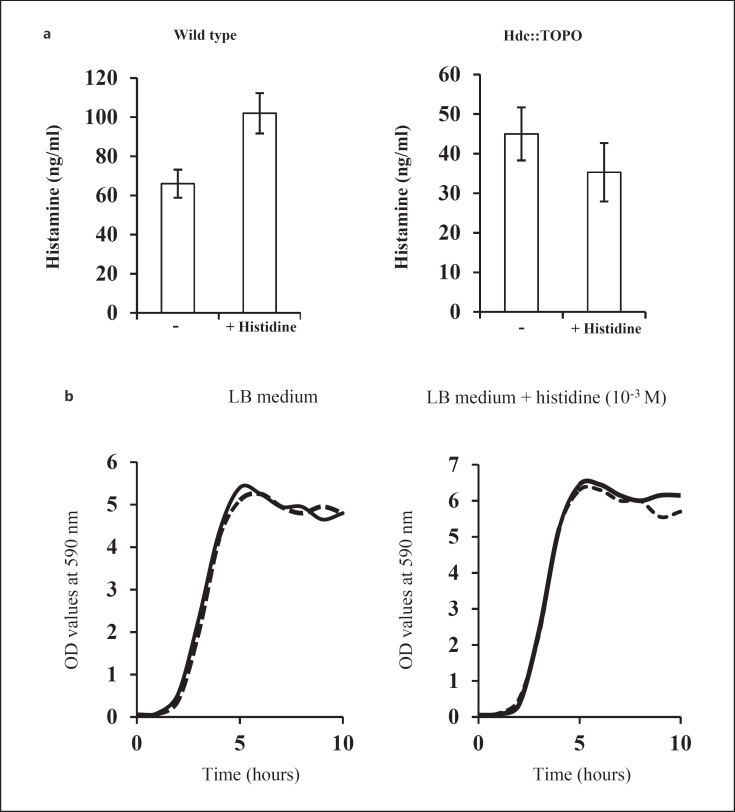
WT *A. baumannii* ATCC 17978, but not hdc::TOPO mutant, produces histamine. **a** *A. baumannii* WT or hdc::TOPO mutant strains were inoculated in LB medium and grown in a glass flask under orbital rotation at 37°C in the absence or presence of histidine (10^−3^ M). After 18 h, aliquots of bacteria were collected and spun down (2,500 *g*, 10 min). The resulting supernatants were transferred to 1.5 mL Eppendorf tubes and aliquots were diluted, and then used for the quantification of histamine by ELISA using the protocol provided by the manufacturer. The data are expressed as mean values ± SD of triplicates. **b** A culture of overnight grown *A. baumannii* ATCC 17978 or hdc::TOPO mutant were diluted in LB broth and adjusted to an OD value of 0.2 at 600 nm. The samples were then diluted 1/100 in LB medium and 10 mL of each mixture was transferred to 250 mL flasks. The flasks were put in an orbital shaker at 37°C. After each hour, an aliquot is taken out from the flask, diluted in LB medium, and turbidimetry at 600 nm was read. The OD values versus time are plotted. Wild type, dashed line; hdc::TOPO mutant, solid line.

We found that addition of histidine (10^−3^ M) to the LB medium, in which the WT *A. baumannii* strain was inoculated, led to ∼1.7-fold increase of the histamine concentration (Fig. 6a, left panel). Other Gram-negative bacteria including *Branhamella catarrhalis, Haemophilus parainfluenzae*, and *Pseudomonas aeruginosa* have also been shown to produce histamine in histidine-enriched growth medium [11]. In contrast, no such increase in histamine concentration was found following inoculation of the hdc::TOPO mutant (Fig. 6a, right panel). As a control, we showed that both the hdc::TOPO mutant and the WT strain have similar growth rates in LB medium regardless of whether or not histidine was added (Fig. 6b). Thus, the lack of histamine production by the hdc::TOPO mutant cannot be attributed to defective growth but to hdc gene disruption.

### Histamine Produced by Gram-Negative Bacteria Is Essential for Pathogenicity

**Fig. 7:**
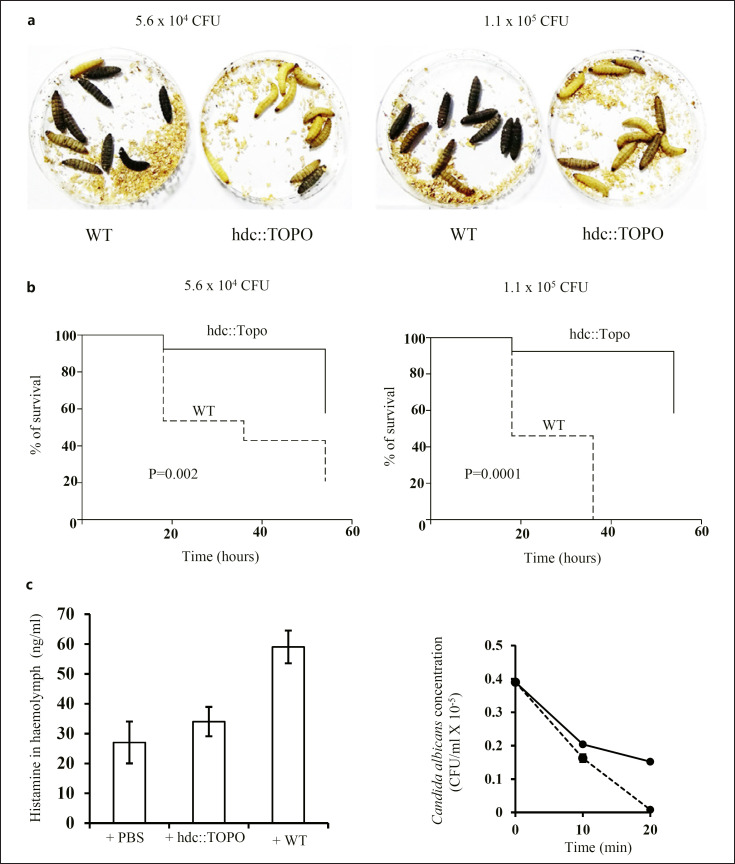
Histamine produced by *A. baumannii* ATCC 17978 in *G. mellonella* larvae contributes to pathogenicity. *G. mellonella* (10 larvae per group) were inoculated, through the proleg, with 5.6 × 10^4^ CFU or 1.1 × 10^5^ CFU of *A. baumannii* ATCC 17978 or hdc::TOPO mutant. Larval survival was recorded after 24 h–54 h. **a** Photograph of larvae 18 h postinfection with *A. baumannii* ATCC 17978 or hdc::TOPO mutant with 5.6 × 10^4^ CFU (left panel), or 1.1 × 10^5^ CFU (right panel). **b** Kaplan-Meier survival analysis upon inoculation of *G. mellonella* larvae with *A. baumannii* ATCC 17978 or hdc::TOPO mutant at 5.6 × 10^4^ CFU (left panel), or 1.1 × 10^5^ CFU (right panel). **c** (left panel) *G. mellonella* larvae were inoculated with PBS (controls), 5.6 × 10^4^ CFU of *A. baumannii* ATCC 17978, or hdc::TOPO mutant for 18 h after which six surviving larvae were collected. The haemolymph of the six larvae were pooled together. The concentration of histamine in the cell-free haemolymph was determined by ELISA. The data represent mean values ± SD of triplicate values. **c** (right panel) The haemolymph of ten larvae were pooled and hemocytes (1 × 10^7^) were incubated with *C. albicans* (2 × 10^6^) in the absence (dashed line) or presence (solid line) of histamine (10^−6^ M). After 10–20 min, the cell mixture was subjected to low speed centrifugation and the supernatants, free of hemocytes, were collected. The number of *C. albicans* remaining in the supernatants was determined by colony counting. The data represent the mean ± SD of three experiments performed in triplicates.

We next compared the pathogenicity of both the hdc::TOPO mutant and the WT strain in *G. mellonella* larvae, an *in vivo*model which has been validated to study *A. baumannii* pathogenesis, producing similar results to those obtained from mammalian studies [28]. We found that inoculation of 5.6 × 10^4^ CFU or 1.1 × 10^5^ CFU of the hdc::TOPO mutant in the larvae did not lead to significant death over a 55 h time course. In contrast, inoculation of similar doses of the WT strain led to rapid death of the larvae. After 48 h following inoculation of 1.1 × 10^5^ CFU of the wild strain, all larvae died whereas, in contrast, with the inoculation of a similar dose of hdc::TOPO mutant, the survival rate was about 70% after 55 h (*p* < 0.0001) (Fig. 7a, b).

In parallel, we measured the concentration of histamine in the haemolymph of *G. mellonella* larvae following inoculation of the vehicle (PBS), the WT strain, or the hdc::TOPO mutant (5.6 × 10^4^ CFU). To this end, we collected the haemolymph of six surviving larvae and determined the concentration of histamine in the pooled, cell-free haemolymph. We found that the concentration of histamine in the haemolymph was ∼60 ng/mL when the larvae had been inoculated with the WT strain, which corresponds to a ∼2-fold increase over basal histamine levels (haemolymph collected after PBS inoculation). In contrast, inoculation of the larvae with the hdc::TOPO mutant did not lead to augmented basal histamine concentration in the haemolymph (∼32 ng/mL) (Fig. 7c, left panel). This result shows that histamine produced by the microorganisms plays a major role in pathogenicity and raises the question of whether histamine regulates the capacity of the immune cells of the host to clear bacteria.

### Histamine Regulates the Capture and Killing of Microorganisms by Hemocytes

The haemolymph of *G. mellonella* larvae is composed of at least six different types of hemocytes, among which granulocytes and plasmatocytes are the most abundant phagocytic cells [29]. Hemocytes from *G. mellonella* capture and kill microorganisms by a mechanism similar to the mechanism used by human neutrophils [30].

We then investigated whether histamine also regulated the ability of hemocytes to capture and/or kill microorganisms. To this end, hemocytes were collected from a pool of ten larvae and incubated with opsonized *Candida albicans (C. albicans)* cells, in the absence or presence of histamine (10^−6^ M). We next measured the kinetic of *C. albicans* cell capture by hemocytes. To do this, we incubated hemocytes with opsonized *C. albicans* cells for different time periods (10–20 min), after which the hemocytes were pelleted, and the amount of *C. albicans* cells remaining in the supernatant (extracellular medium) was determined by colony counting.

We found that the number of *C. albicans* cells in the extracellular medium decreased linearly with time in control hemocytes. After 20 min, we could not detect any *C. albicans* cells left in the extracellular medium meaning that all yeast cells were captured. In contrast, a delay in the capture of *C. albicans* cells was observed when hemocytes were exposed to histamine (10^−6^ M). This is illustrated by the fact that half of the yeast cells were captured after 20 min incubation (Fig. 7c, right panel). Thus, histamine produced in *G. mellonella* larvae by Gram-negative bacteria may block hemocyte phagocytosis, and this may explain, at least in part, the high mortality rate of the larvae in response to inoculation with *A. baumannii* WT but not hdc::TOPO mutant.

**Fig. 8:**
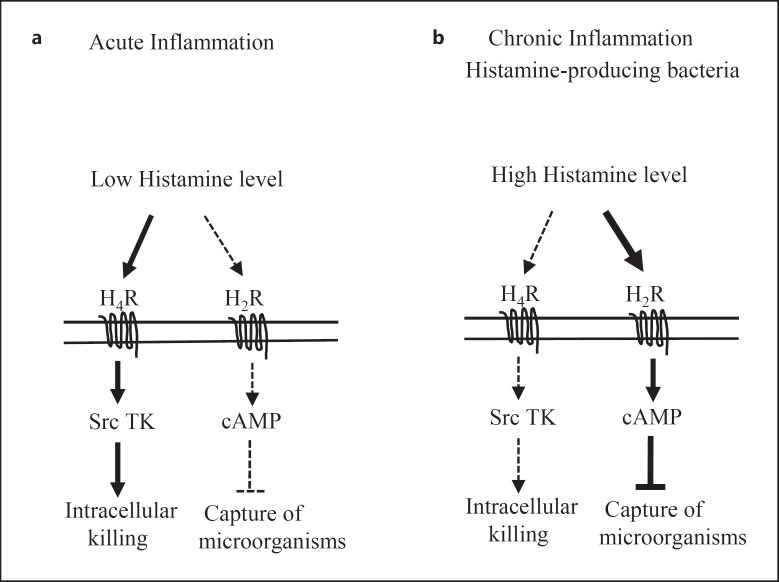
The H_4_R and the H_2_R have different roles in human neutrophils. During acute infection, the concentration of histamine is low leading to engagement of the H_4_R, but not the H_2_R. Engagement of the H_4_R accelerates intracellular killing of microorganisms and this is achieved through coupling of the H_4_R to Src family tyrosine kinases and activation of inflammatory functions. Later on, the concentration of histamine rises due to the abundance of neutrophils. The H_2_R is then engaged and the cAMP/PKA pathway is activated, leading to inhibition of Mac-1-dependent neutrophil functions including capture of microorganisms. In the context of chronic infection, Gram-negative bacteria, by producing histamine, may impair neutrophil functions through engagement of the H_2_R. For more details, please refer to the Discussion section.

In Figure 8, we propose a model explaining the roles of the H_2_R and H_4_R in the regulation of the capture and killing of microorganisms by neutrophils. For more details, please refer to the discussion section.

## Discussion/Conclusion

In this study, we investigated the contribution of the H_2_R and the H_4_R in the regulation of the capture and/or killing of microorganisms by neutrophils. We found that stimulation of neutrophils with histamine (10^−9^ M), a concentration which activates the H_4_R, but not the H_2_R, had no effect on the capture by neutrophils of serum-opsonized *E. coli* but significantly accelerated the intracellular killing of *E. coli*. Intracellular killing of microorgansims is dependent upon activation of neutrophil inflammatory functions: ROS is produced inside the phagosomes and antimicrobials present in granules are released inside the phagosomes to kill engulfed microorganisms. In neutrophils, activation of inflammatory functions, in response to bacteria-derived formyl-peptides, requires the Src family tyrosine kinases including Hck and Fgr [23, 24, 25]. This was well demonstrated by using hck^−/−^fgr^−/−^ mice. Neutrophils isolated from hck^−/−^fgr^−/−^ mice show absence of ROS production and degranulation, in response to the chemotactic peptide fMLP [23]. Accordingly, we investigated whether the H_4_R, similarly to fMLP receptors, was also coupled to the Src family tyrosine kinases. We found that nanomolar concentrations of histamine induced tyrosine phosphorylation of proteins in both human neutrophils and the neutrophil-like PLB-985 cells, such phosphorylation events were blocked by the Src family tyrosine kinase inhibitor PP2. Furthermore, the pattern of tyrosine phosphorylated proteins, obtained in response to histamine, was very similar to the one obtained with fMLP. We further proved that the H_4_R, but not the H_2_R, is coupled to the Src family tyrosine kinases by showing that histamine-induced tyrosine phosphorylation of proteins was totally abolished by the H_4_R antagonist JNJ 7777120, but not by the H_2_R antagonist famotidine. Therefore, the coupling of the H_4_R to the Src family tyrosine kinase pathway explains how this receptor controls activation of intracellular killing of microorganisms by neutrophils.

In contrast, at high concentration, histamine (10^−6^ M) significantly delayed the capture of *E. coli* by neutrophils and reduced the accumulation of zymosan particles inside acidic mature phagosomes indicating that engagement of the H_2_R impairs Mac-1, the receptor binding complement-opsonized microorganisms and involved in pathogen clearance [31]. Since TNF-α augments expression of Mac-1 on the cell membrane and converts Mac-1 into an active ligand binding conformation [32], reduced capture of *E. coli* in response to histamine (10^−6^ M) could be due to inhibition by the diamine of TNF-α-induced activation of Mac-1. In favor of this scenario, it was shown that histamine decreases the binding of neutrophils to surfaces coated with complement and inhibits the ability of Mac-1 to cluster to the plasma membrane thereby preventing Mac-1 binding ligands [33]. Also supporting this is the fact that the PKA inhibitor H-89 reversed the capacity of histamine (10^−6^ M) to delay bacterial capture and inhibition of PKA activity has been linked to activation of neutrophil β2 integrins [34]. In contrast, we found that histamine had no effect on basal as well as TNF-α-induced neutrophil adhesion on plates coated with fibrinogen, a ligand for Mac-1 [16]. Therefore, it is plausible that histamine controls both ligand-induced activation of Mac-1 and signals downstream of Mac-1 activation controlling the dynamic of the actin-based cytoskeleton, a key feature for the formation of the phagocytic cup and internalization of the microorganisms [35].

We next investigated whether the H_2_R was coupled to the cAMP/PKA pathway because this pathway has been shown to prevent activation of Mac-1 in neutrophils by reducing myosin light-chain phosphorylation [34]. We provided support for a coupling of the H_2_R to the cAMP/PKA pathway by showing that histamine, at high, but not low concentrations, induced phosphorylation of VASP on Ser 157 (used as readout to measure indirectly activation of PKA), an effect totally abolished by the H_2_R antagonist famotidine, but not by the H_4_R antagonist JNJ 7777120. The mechanism by which the cAMP/PKA pathway impairs Mac-1 activation is not known but may involve, at least in part, VASP phosphorylation on Ser 157. Indeed, we showed that neutrophils isolated from VASP^−/−^ mice exhibited constitutive activation of Rap1, the GTPase controlling the conformational activation of Mac-1, and this was associated with augmented adhesion on fibrinogen, a ligand for Mac-1 [12]. Augmented Rap1 activation in neutrophil from VASP^−/−^ mice may be explained by the fact that when VASP is not expressed, inhibition of Rap1 through PKA-dependent phosphorylation of VASP on Ser 157 is lost. We also provided evidence for a role of PKA-dependent phosphorylation of VASP on Ser 157 in the inhibition of ligand-induced activation of Rap1 and integrins in platelets [36].

Mac-1 is not only the receptor binding complement-opsonized microorganisms, it is also the dominant integrin controlling adhesion and migration of neutrophils to the site of inflammation as well as activation of adhesion-dependent inflammatory functions [37]. Therefore, histamine by binding and activating the H_2_R, may also play a role in the resolution of inflammation. Such a role of the H_2_R is well supported by findings showing that histamine impairs fMLP-induced superoxide production and degranulation [38], effects which are reversed by H_2_R antagonists. Histamine also inhibits fMLP-induced leukotriene biosynthesis, an effect reversed by H_2_R antagonists [39]. Leukotrienes, among which LTB4, are molecules amplifying formyl peptide-mediated neutrophil polarization and chemotaxis [40].

We next addressed the role of histamine produced by bacteria during infection. We found that *G. mellonella* larvae inoculated with a high CFU of the hdc::TOPO mutant have a low mortality rate. In contrast, larvae inoculated with the WT strain did not survive. This result is significant because it demonstrates that it is the histamine produced by the bacteria in the host that contributes to pathogenicity. There are at least two ways by which histamine could protect the invading pathogen in the host. Histamine could favor bacterial growth, survival, or virulence. Such scenario could be envisioned for bacteria such as *Pseudomonas aeruginosa* strains which express the histamine receptor TlpQ controlling bacterial migration and accumulation at sites of infection modulating in turn expression of virulent factors [41].

A recent study has shown that disruption of the basG gene (which encodes a HDC), in *A. baumannii* ATCC 17978, interrupts the acinetobactin biosynthetic pathway as histamine is an essential precursor molecule to the biosynthesis of this siderophore [42]. These acinetobactin biosynthetic mutants have reduced capacity for acquiring iron from host sources and therefore have a reduced growth *in vitro*. However, we noted that the difference between the WT and basG gene-deficient mutants, in terms of *in vitro* growth under iron restriction, is relatively modest, and this is explained by the fact that basG gene-deficient mutants have other siderophores structurally different from acitenobactin, with similar iron-capturing functions. In contrast, acinetobactin biosynthetic mutants are severely attenuated for survival and proliferation in various tissues, organs or fluids in a mouse bacteremia model, hence high mouse survival rates. In light of our data showing that histamine regulates the capture and killing of microorganisms by neutrophils, it is plausible that the high survival rate of mice infected with basG gene-deficient mutants cannot be solely explained by a lack of acinetobactin synthesis but could be largely due to augmented clearance of these bacteria by neutrophils.

In support of this hypothesis, we found that infection of *G. mellonella* larvae with *A. baumannii* WT lead to augmented histamine concentration in the haemolymph of the larvae. In contrast, a similar infection with *A. baumannii* hdc::TOPO mutant, did not. This implies that hemocytes are exposed to higher concentrations of histamine (in the micromolar range) during the period of infection. Such exposure to histamine may compromise the capacity of the hemocytes to capture microorganisms, similarly to what we found in human neutrophils. We confirmed this assumption by showing that incubation of hemocytes from *G. mellonella* with histamine (10^−6^M) significantly delayed the capture of *C. albicans in vitro*. In keeping with our finding, treatment of hemocytes of the tunicate *Styela plicata* with histamine reduced their phagocytic capacity *in vitro* and ex *in vivo* following systemic injection of histamine into the organism [8]. Thus, by producing histamine in the host, bacteria may delay their capture and killing by hemocytes and this would explain, at least in part, the high mortality rate of *G. mellonella* inoculated with the WT strain and the high survival rate of *G. mellonella* inoculated with the hdc::TOPO mutant. Colonization of the airways by Gram-negative respiratory bacteria is a hallmark of acute exacerbations of chronic bronchitis, cystic fibrosis, and pneumonia in mammals. Incubation of sputum samples from patients with exacerbations of chronic bronchitis or cystic fibrosis lead to augmented histamine levels [43], indicating that the airways of these patients contain bacteria synthesizing histamine. Such synthesis is made possible because some Gram-negative bacteria possess a pyridoxal-dependent hdc gene in their genome [27]. Furthermore, high histamine concentrations are found in the sputum samples of patients with chronic obstructive bronchitis [44]. Thus, respiratory bacteria contribute to sputum histamine in infective lung diseases. In light of our data showing augmented histamine concentration in the haemolymph of *G. mellonella* in response to *A. baumannii* inoculation and its association with reduced larvae survival, it is plausible that accumulation of histamine in the airways of patients with chronic airways infectious diseases compromises airways neutrophil functions.

Beside a direct regulatory role of histamine on neutrophil phagocytosis, histamine has also been shown to regulate neutrophil recruitment to the site of inflammation. For example, the clearance of *E. coli* is augmented in mice deficient in histamine synthesis (HDC^−/−^ mice) and this was associated with increased number of neutrophils recruited to the peritoneal cavity [45]. In contrast, in a model of murine experimental colitis, histamine produced by tissue resident mast cells controls H_4_R-dependent production of neutrophil chemokines by mucosal epithelial cells and neutrophil invasion of mucosa leading to severe inflammation [46]. Similarly, in a HDC^−/−^ mouse model of lung *Mycobacterium tuberculosis* infection, histamine plays a role in enhancing pulmonary neutrophilia through production of the proinflammatory cytokines IL-6 and TNF-α [47]. Thus, conflicting data indicate histamine has both proinflammatory and anti-inflammatory activity [48].

In summary (Fig. 8), we propose that histamine is produced by neutrophils during periods of infection to accelerate the killing of microorganisms. This effect of histamine is controlled by the H_4_R, a receptor activated by low concentrations of histamine. Engagement of the H_4_R leads to the activation of the Src family tyrosine kinase pathway controlling neutrophil inflammatory functions required for pathogen killing. At the end of the inflammatory process, the concentration of histamine rises due to the large number of infiltrated neutrophils. The H_2_R is then engaged, and the cAMP/PKA pathway is activated, leading to the inhibition of Mac-1-dependent functions including capture of complement opsonized microorganisms, inflammatory functions, and recruitment of neutrophils.

However, in the context of chronic infections or high CFU of pathogens during the acute phase of infection, bacteria-producing histamine may hijack this regulatory mechanism by maintaining high levels of histamine to impair neutrophil phagocytosis by engaging the H_2_R and possibly desensitizing the H_4_R. This scenario could explain, at least in part, why in chronic lung diseases (cystic fibrosis, chronic obstructive pulmonary disease), there is persistence of respiratory bacterial infections despite the abundance of neutrophils in the airways which are expected to kill the pathogens but fail to do so [49].

## Statement of Ethics

The work was conducted in accordance with the World Medical Association Declaration of Helsinki. The study protocol was approved by the Ethics Committee of the School of Medicine, Dentistry, and Biomedical Sciences, Queen's University Belfast (reference number: MHLS 20-146). Blood donors have given their written informed consent to participate in this study.

## Conflict of Interest Statement

The authors declare no conflict of interest.

## Funding Sources

This work was financially supported by an MRC-Industry Asset Sharing Initiative grant (ref: MR/R005915/1) from the Medical Research Council UK (To KD), and by a grant from Northern Ireland Chest Heart and Stroke (KD & CR).

## Author Contributions

Karim Dib, Amal El Banna, Clara Radulescu, and Gerard Sheehan performed experimental work; Guillermo Lopez Campos contributed to the theoretical design of the *A. baumannii* mutants; Karim Dib and Kevin Kavanagh designed the study and supervised the work; Karim Dib wrote the manuscript; all the authors read the manuscript prior to submission.

## Data Availability Statement

The data will be made publicly available without any restrictions. For further information, please contact the corresponding author Dr. Karim Dib.

## References

[1] Kolaczkowska E, Kubes P (2013). Neutrophil recruitment and function in health and inflammation. Nat Rev Immunol.

[2] Ueda T, Rieu P, Brayer J, Arnaout AA (1994). Identification of the complement iC3b binding site in the beta 2 integrin CR3 (CD11b/CD18). Proc Natl Acad Sci U S A.

[3] Tanaka S, Deai K, Konomi A, Takahashi K, Yamane H, Sugimoto Y (2004). Expression of L-histidine decarboxylase in granules of elicited mouse polymorphonuclear leukocytes. Eur J Immunol.

[4] Xu X, Zhang D, Zhang H, Wolters PJ, Killeen NP, Sullivan BM (2006). Neutrophil histamine contributes to inflammation in mycoplasma pneumonia. J Exp Med.

[5] Xu X, Zhang H, Song Y, Lynch SV, Lowell CA, Wiener-Kronish JP (2012). Strain-dependent induction of neutrophil histamine production and cell death by *Pseudomonas aeruginosa*. J Leukoc Biol.

[6] Smuda C, Wechsler JB, Bryce PJ (2011). TLR-induced activation of neutrophils promotes histamine production via a PI3 kinase dependent mechanism. Immunol Lett.

[7] Alcañiz L, Vega A, Chacón P, El Bekay R, Ventura I, Aroca R (2013). Histamine production by human neutrophils. FASEB J.

[8] García-García E, Gómez-González NE, Meseguer J, García-Ayala A, Mulero V (2014). Histamine regulates the inflammatory response of the tunicate *Styela plicata*. Developmental Comp Immunol.

[9] Ennis M, Ciz M, Dib K, Friedman S, Gangwar RS, Gibbs BF, Stark H (2013). Histamine receptors and inflammatory cells. Histamine H4 receptor: a novel drug target in immunoregulation and inflammation.

[10] Thurmond RL, Gelfand E, Dunford P (2008). The role of histamine H1 and H4 receptors in allergic inflammation: the search for new antihistamines. Nat Rev Drug Discov.

[11] Devalia JL, Grady D, Harmanyeri Y, Tabaqchali S, Davies RJ (1989). Histamine synthesis by respiratory tract micro-organisms: possible role in pathogenicity. J Clin Pathol.

[12] Deevi RK, Koney-Dash M, Kissenpfennig A, Johnston JA, Schuh K, Walter U (2010). Vasodilator-stimulated phosphoprotein regulates inside-out signaling of beta2 integrins in neutrophils. J Immunol.

[13] Pivot-Pajot C, Chouinard FC, El Azreq MA, Harbour D, Bourgoin SG (2010). Characterisation of degranulation and phagocytic capacity of a human neutrophilic cellular model, PLB-985 cells. Immunobiology.

[14] Hampton MB, Vissers MCM, Winterbourn CC (1994). A single assay for measuring the rates of phagocytosis and bacterial killing by neutrophils. J Leukoc Biol.

[15] Vaudaux P, Waldvogel FA (1979). Gentamicin antibacterial activity in the presence of human polymorphonuclear leukocytes. Antimicrob Agents Chemother.

[16] Dib K, Perecko T, Jenei V, McFarlane C, Comer D, Brown V (2014). The histamine H4 receptor is a potent inhibitor of adhesion-dependent degranulation in human neutrophils. J Leukoc Biol.

[17] Aranda J, Poza M, Pardo BG, Rumbo S, Rumbo C, Parreira JR (2010). A rapid and simple method for constructing stable mutants of *Acinetobacter baumannii*. BMC Microbiol.

[18] Fallon J, Kelly J, Kavanagh K (2012). Galleria mellonella as a model for fungal pathogenicity testing. Methods Mol Biol.

[19] Morgan D, Capasso M, Musset B, Cherny VV, Ríos E, Dyer MJS (2009). Voltage-gated proton channels maintain pH in human neutrophils during phagocytosis. Proc Natl Acad Sci U S A.

[20] Deryugina E, Zajac EI, Juncker-Jensen A, Kupriyanova TA, Welter L, Quigley JP (2014). Tissue-infiltrating neutrophils constitute the major in vivo source of angiogenesis-inducing MMP-9 in the tumor microenvironment. Neoplasia.

[21] Dash-Koney M, Deevi RK, McFarlane C, Dib K (2011). Exchange protein directly activated by cAMP 1 (Epac1) is expressed in human neutrophils and mediates cAMP-dependent activation of the monomeric GTPase Rap1. J Leukoc Biol.

[22] Eckert RE, Jones SL (2007). Regulation of VASP serine 157 phosphorylation in human neutrophils after stimulation by a chemoattractant. J Leukoc Biol.

[23] Fumagalli L, Zhang H, Baruzzi A, Lowell CA, Berton G (2007). The Src family kinases Hck and Fgr regulate neutrophil responses to N-formyl-methionyl-leucyl-phenylalanine. J Immunol.

[24] Berton G, Mócsai A, Lowell CA (2005). Src and Syk kinases: key regulators of phagocytic cell activation. Trends Immunol.

[25] Mócsai A, Jakus Z, Vántus T, Berton G, Lowell CA, Ligeti E (2000). Kinase pathways in chemoattractant-induced degranulation of neutrophils: the role of p38 Mitogen-activated protein kinase activated by Src family kinases. J. Immunol.

[26] Rajagopal S, Shenoy SK (2018). GPCR desensitization: acute and prolonged phases. Cell Signal.

[27] Kamath AV, Vaaler GL, Snell EE (1991). Pyridoxal phosphate-dependent histidine decarboxylases. Cloning, sequencing, and expression of genes from *Klebsiella planticola* and *Enterobacter aerogenes* and properties of the overexpressed enzymes. J Biol Chem.

[28] Peleg AY, Jara S, Monga D, Eliopoulos GM, Moellering RC, Mylonakis E (2009). Jr *Galleria mellonella* as a model system to study *Acinetobacter baumannii* pathogenesis and therapeutics. Antimicrob Agents Chemother.

[29] Ratcliffe NA, Gagen SJ (1977). Studies on the in vivo cellular reactions of insects: an ultrastructural analysis of nodule formation in *Galleria mellonella*. Tissue Cell.

[30] Bergin D, Reeves EP, Renwick J, Wientjes FB, Kavanagh K (2005). Superoxide production in *Galleria mellonella* hemocytes: identification of proteins homologous to the NADPH oxidase complex of human neutrophils. Infect Immun.

[31] Torres-Gomez A, Cabañas C, Lafuente EM (2020). Phagocytic integrins: activation and signaling. Front Immunol.

[32] Montecucco F, Steffens S, Burger F, Da Costa A, Bianchi G, Bertolotto M (2008). Tumor necrosis factor-alpha (TNF-alpha) induces integrin CD11b/CD18 (Mac-1) up-regulation and migration to the CC chemokine CCL3 (MIP-1alpha) on human neutrophils through defined signalling pathways. Cell Signal.

[33] Francis JW, Todd R, Boxer LA, Petty HR (1991). Histamine inhibits cell spreading and C3bi receptor clustering and diminishes hydrogen peroxide production by adherent human neutrophils. J Cell Physiol.

[34] Chilcoat CD, Sharief Y, Jones SL (2008). Tonic protein kinase A activity maintains inactive beta2 integrins in unstimulated neutrophils by reducing myosin light-chain phosphorylation: role of myosin light-chain kinase and Rho kinase. J Leukoc Biol.

[35] Groves E, Dart AE, Covarelli V, Caron E (2008). Molecular mechanisms of phagocytic uptake in mammalian cells. Cell Mol Life Sci.

[36] Benz PM, Laban H, Zink J, Günther L, Walter U, Gambaryan S (1186). Vasodilator-Stimulated Phosphoprotein (VASP)-dependent and -independent pathways regulate thrombin-induced activation of Rap1b in platelets. Cell Commun Signal.

[37] Ley K, Laudanna C, Cybulsky MI, Nourshargh S (2007). Getting to the site of inflammation: the leukocyte adhesion cascade updated. Nat Rev Immunol.

[38] Seligmann BE, Fletcher MP, Gallin JI (1983). Histamine modulation of human neutrophil oxidative metabolism, locomotion, degranulation, and membrane potential changes. J Immunol.

[39] Flamand N, Plante H, Picard S, Laviolette M, Borgeat P (2004). Histamine-induced inhibition of leukotriene biosynthesis in human neutrophils: involvement of the H2 receptor and cAMP. Br J Pharmacol.

[40] Afonso PV, Janka-Junttila M, Lee YJ, McCann CP, Oliver Charlotte CM, Aamer KA (2012). LTB4 is a signal-relay molecule during neutrophil chemotaxis. Dev Cell.

[41] Corral-Lugo A, Matilla MA, Martín-Mora D, Silva Jiménez HS, Mesa Torres NM, Kato J (2018). High-affinity chemotaxis to histamine mediated by the TlpQ chemoreceptor of the human pathogen *Pseudomonas aeruginosa*. mBio.

[42] Sheldon JR, Skaar EP (2020). *Acinetobacter baumannii* can use multiple siderophores for iron acquisition, but only acinetobactin is required for virulence. PLoS Pathog.

[43] Sheinman BD, Devalia JL, Crook SJ, Davies RJ (1986). De novo generation of histamine in sputum and the effect of antibiotics. Agents Actions.

[44] Zimmermann I, Bugalho de Almeida AA, Ulmer WT (1987). Histamine content of the sputum of patients with obstructive bronchitis. Respiration.

[45] Hori Y, Nihei Y, Kurokawa Y, Kuramasu A, Makabe-Kobayashi Y, Terui T (2002). Accelerated clearance of *Escherichia coli* in experimental peritonitis of histamine-deficient mice. J Immunol.

[46] Wechsler JB, Szabo A, Hsu CL, Krier-Burris RA, Schroeder HA, Wang MY (2018). Histamine drives severity of innate inflammation via histamine 4 receptor in murine experimental colitis. Mucosal Immunol.

[47] Carlos D, Fremond C, Samarina A, Vasseur V, Maillet I, Ramos S (2009). Histamine plays an essential regulatory role in lung inflammation and protective immunity in the acute phase of *Mycobacterium tuberculosis* infection. Infect Immun.

[48] Jutel M, Watanabe T, Akdis M, Blaser K, Akdis CA (2002). Immune regulation by histamine. Curr Opin Immunol.

[49] Morris MR, Doull IJ, Dewitt S, Hallett MB (2005). Reduced iC3b-mediated phagocytotic capacity of pulmonary neutrophils in cystic fibrosis. Clin Exp Immunol.

